# Engineering Oleaginous Yeast as the Host for Fermentative Succinic Acid Production From Glucose

**DOI:** 10.3389/fbioe.2019.00361

**Published:** 2019-11-27

**Authors:** Mahsa Babaei, Kanchana Rueksomtawin Kildegaard, Aligholi Niaei, Maryam Hosseini, Sirous Ebrahimi, Suresh Sudarsan, Irini Angelidaki, Irina Borodina

**Affiliations:** ^1^Department of Chemical & Petroleum Engineering, University of Tabriz, Tabriz, Iran; ^2^The Novo Nordisk Foundation Center for Biosustainability, Technical University of Denmark, Lyngby, Denmark; ^3^Department of Chemical Engineering, Faculty of Engineering, Azarbaijan Shahid Madani University, Tabriz, Iran; ^4^Biotechnology Research Center, Faculty of Chemical Engineering, Sahand University of Technology, Tabriz, Iran; ^5^Department of Environmental Engineering, Technical University of Denmark, Lyngby, Denmark

**Keywords:** metabolic engineering, succinic acid, *SDH1*, *Yarrowia lipolytica*, fed-batch fermentation

## Abstract

Oleaginous yeast *Yarrowia lipolytica* is a prospective host for production of succinic acid. The interruption of tricarboxylic acid cycle through succinate dehydrogenase gene (*SDH*) deletion was reported to result in strains incapable of glucose utilization and this ability had to be restored by chemical mutation or long adaptive laboratory evolution. In this study, a succinate producing strain of *Y. lipolytica* was engineered by truncating the promoter of *SDH1* gene, which resulted in 77% reduction in *SDH* activity but did not impair the ability of the strain to grow on glucose. The flux toward succinic acid was further improved by overexpressing the genes in the glyoxylate pathway and the oxidative TCA branch, and expressing phosphoenolpyruvate carboxykinase from *Actinobacillus succinogenes*. A short adaptation on glucose reduced the lag phase of the strain and increased its tolerance to high glucose concentrations. The resulting strain produced 7.8 ± 0.0 g/L succinic acid with a yield of 0.105 g/g glucose in shake flasks without pH control, while mannitol (11.8 ± 0.8 g/L) was the main by-product. Further investigations showed that mannitol accumulation was caused by low pH stress and buffering the fermentation medium eliminated mannitol formation. In a fed-batch bioreactor in mineral medium at pH 5, at which point according to K_a_ values of succinic acid, the major fraction of product was in acidic form rather than dissociated form, the strain produced 35.3 ± 1.5 g/L succinic acid with 0.26 ± 0.00 g/g glucose yield.

## Introduction

Succinic acid (C_4_H_6_O_4_) is a potential platform chemical with a wide range of applications in food, pharmacy, biopolymers, coatings, green solvents, and plasticizers (Ahn et al., 2016). Succinic acid can be chemically converted into other value-added products, as 1,4-butanediol, γ-butyrolactone, *N*-methyl-2-pyrrolidone, tetrahydrofurane, and 2-pyrrolidone (Pateraki et al., 2016). Succinic acid market demand comprised 50,000 metric tons in 2016 and is expected to double by 2025 (Chinthapalli et al., 2018). The total plant capacity for production of succinic acid by fermentation is about 64,000 tons per year (BioAmber, Myriant—now GC Innovation America, Reverdia and Succinctly).

Various microbes have been engineered for production of succinate by fermentation. The highest volumetric rates have been reported for rumen bacterium *Mannheimia succiniciproducens*. The group of Sang Yup Lee reported titer of 78.4 g/L with yield of 1.64 mol/mol glucose and overall volumetric productivity of 6.02 g/L/h on a mixed feed of glycerol and sucrose (Lee et al., 2016). Bacterial fermentations typically require neutral pH and result in succinate salt. In the downstream processing, succinate must be acidified into succinic acid, generating large amounts of by-product, such as gypsum (Sauer et al., 2008). This can be avoided if the fermentation is to be carried out at low pH.

Many yeasts are tolerant to low pH, some species as *Y. lipolytica* can grow at pH as low as 3.5 (Mironczuk et al., 2016). Considering the pKa of succinic acid of 4.2 and 5.6 at 25°C (Dean, 1999), at pH 3.5 more than 80% of succinic acid would be in protonated form. *Saccharomyces cerevisiae* has been engineered for succinic acid production by Reverdia (joint venture of DSM and Roquette), which required, among other modifications, a deletion of pyruvate decarboxylases to prevent ethanol fermentation. In 2013, BioAmber/Mitsui switched from *Escherichia coli* as host microorganism to the yeast *Candida krusei*, a low pH-tolerant strain discovered by Cargill (Jansen and van Gulik, 2014).

In the past decade, non-conventional yeast *Y. lipolytica* has become relatively well-amenable for genetic manipulation and it has been engineered for commercial production of polyunsaturated fatty acids (DuPont) and lipids (Novogy, acquired by Total). This yeast has a long record of safe use, since it's been used as food additive and supplement in 80's and has undergone extensive toxicity tests for this purpose (Zinjarde, 2014). Some strains of *Y. lipolytica* are native overproducers of tricarboxylic acid (TCA) cycle acids: citric (Cavallo et al., 2017), isocitric (Kamzolova et al., 2013) and α-ketoglutaric (Guo et al., 2016), which renders the species a potentially attractive host for the production of another TCA cycle acid, namely succinic acid. Kamzolova et al have utilized the capability of this yeast to secrete high titers of α-ketoglutaric acid for succinic acid production in a two-step process. The strain VKMY-2412 was engineered to produce 88.7 g/L α-ketoglutaric acid, which was subsequently decarboxylized chemically to succinic acid in the presence of H_2_O_2_ (Kamzolova et al., 2014a,b).

A number of studies on engineering *Y. lipolytica* for direct succinic acid production have been published (Table 1).The common feature of all the reported strains is reduced succinate dehydrogenase activity, to decrease the succinate conversion into fumarate in the TCA cycle. The succinate dehydrogenase (*SDH*) complex in *Y. lipolytica* includes five subunits, flavoprotein subunit *YALI0D11374g* (*SDH1*), iron-sulfur subunit *YALI0D23397g* (*SDH2*), cytochrome b560 subunit *YALI0E29667g* (*SDH3*), membrane anchor subunit *YALI0A14784g* (*SDH4*), and succinate dehydrogenase assembly factor 2 *YALI0F11957g* (*SDH5*). The early study from Yuzbashev et al. (2010) reported a *Y. lipolytica* strain with deletion of *SDH2* gene. The deletion of *SDH2* gene impaired the utilization of glucose, while glycerol utilization was intact. The disruption of TCA cycle through inhibition of the conversion of succinate to fumarate by *SDH* gene deletion leads to insufficient regeneration of reducing equivalents (FADH2) that results in less ATP synthesis through oxidative phosphorylation in yeast cells. In addition, the export of produced succinic acid is an energy dependent process that aggravates the ATP deficiency in the *SDH*-deleted mutants. The inadequate ATP is considered as the reason for observed loss of the ability of cells to grow on glucose after *SDH* deletion. However, switching the carbon source from glucose to glycerol metabolism, on the other hand, generates 3 ATP molecules more than glucose that makes the cell growth and succinic acid production feasible even at low pH (Yuzbashev et al., 2011).

**Table 1:** Overview of production of succinic acid by *Yarrowia lipolytica*.

**Strain**	**Genotype**	**Cultivation**	**Medium and carbon source**	**Succinic acid performance metrics**	**Reference**
Y-3314	High viability mutant of ^a^PO1f *^b^ΔSDH2::*^c^*URA3*	Shake flask	Complex with glycerol (^d^YPG) without pH control. Final pH 3.2	17.4 g/L in 7 days 0.87 g/g glycerol^*^	Yuzbashev et al., 2010
Y-4215	Chemical mutagenesis and adaptive evolution of Y-3314 in low pH chemostat (840 h)	Bioreactor	Mineral with glucose, without pH control. Final pH 2.7	50.2 g/L in 54 h, 0.43 g/g glucose	Yuzbashev et al., 2016
VKPM Y-3753	Multistage mutation including chemical mutagenesis and selection	Test Tube (5 mL)	Complex with glucose (^e^YPD), without pH control. Final pH 3.	60 g/L in 96 h, 0.57 g/g glucose^*^	Sineokii et al., 2012
		Bioreactor	Mineral with glucose, without pH control. Final pH 3.65	55.3 g/L in 48 h, 0.34 g/g glucose	Bondarenko et al., 2017
H222-AZ2	^f^H222 ^g^*POT1-SDH2 URA3*	Bioreactor	Mineral with glycerol, pH controlled at 5.0	25 g/L in 165 h, 0.26 g/g glycerol	Jost et al., 2014
PGC01003	PO1f *ΔSDH5::URA3*	Bioreactor	Complex with crude glycerol (YPG), pH controlled at 6.0	160 g/L in 400 h, 0.40 g/g glycerol^*^	Gao et al., 2016
		^h^isFB Bioreactor	Complex with glycerol (YPG), with pH controlled at 6.0	198.2 g/L in 238 h, 0.42 g/g glycerol^*^	Li et al., 2017
PSA02004	Evolutionary adapted mutant of PGC01003 (21 days)	Bioreactor	Complex with glucose (YPD), pH controlled at 6.0	65.7 g/L in 96 h, 0.50 g/g glucose^*^	Yang et al., 2017
PSA3.0	Evolutionary adapted (62 days) mutant of PSA02004	^h^isFB Bioreactor	Complex with glucose (YPD), pH controlled at 3.0.	18.4 g/L, 0.23 g/g glucose^*^	Li et al., 2018a
PGC202	PGC01003 ^i^(*ΔACH1::loxP ScPCK SCS2*)	Bioreactor	Complex with glycerol (YPG), without pH control. Final pH 3.4	110.7 g/L in 138 h, 0.53 g/g glycerol^*^	Cui et al., 2017
			Complex with glucose (YPD), without pH control. Final pH 5	53.6 g/L in 110 h, 0.61 g/g glucose^*^	Yu et al., 2018
ST8578	Short adaptation (5 days) of W29 ^j^(*SpMAE1 ΔACH1 tPSDH1_95bp SCS2 KGDH MLS MDH ICL AsPCK*)	Bioreactor	Mineral with glucose, pH controlled at 5.0	35.3 g/L in 58 h, 0.26 g /g glucose	This study

a *PO1f, W29 MatA, leu2-270, ura3-302, xpr2-322, axp-2, auxotrophic for Leu and Ura*.

b *SDH, Succinate dehydrogenase gene, subunit indicated after the name of the gene*.

c *URA3, Uracil requiring Orotidine-5'-phosphate decarboxylase*.

d *YPG, Medium with 10 g/L yeast extract, 20 g/L peptone, and glycerol*.

e *YPD, Medium with 10 g/L yeast extract, 20 g/L peptone, and glucose*.

f *H222, W29 MATA ura3-302 ku70Δ-1572*.

g *POT1, 3-ketoacyl-CoA thiolase encoding gene from Y. lipolytica*.

h *isFB, in situ fibrous bed reactor*.

i *ACH1, acetyl-CoA hydrolase, ScPCK: phosphoenolpyruvate carboxykinase from S. cerevisiae, SCS2: succinyl-CoA synthase beta-subunit*.

j *SpMAE1, dicarboxylic acid transporter from Schizosaccharomyces pombe; tPSDH1_95bp, truncated promoter of SDH1 gene to 95 bp; KGDH, α-ketoglutarate dehydrogenase; MLS, malate synthase; MDH, malate dehydrogenase; ICL, isocitrate lyase; AsPCK, phosphoenolpyruvate carboxykinase from Actinobacillus succinogenes*.

* *Yield numbers for complex media can be misleading because other media components are used as carbon sources as well*.

A high viability mutant of the knock-out strain Y-3314 produced 17.4 g/L succinic acid from glycerol in shake flasks. The strain was further improved by chemical mutation and selection, a better performing mutant Y-4215 was identified, producing 50.2 g/L succinic acid on glucose (Yuzbashev et al., 2016). Sineokii et al. patented a strain of *Y. lipolytica* VKPM Y-3753, obtained by multistage mutagenesis using nitrosoguanidine and subsequent two-stage selection. The strain produced up to 60 g/L succinic acid in test tubes from glucose at low pH of 3 in rich medium (Sineokii et al., 2012). Optimization of the cultivation mode of this strain in bioreactor resulted in 55.3 g/L in 48 h produced on mineral medium with glucose without pH control (Bondarenko et al., 2017).

Jost et al. constructed a *Y. lipolytica* strain H222-AZ2 with a 64% reduction in *SDH* activity by replacing the indigenous endogenous promoter of gene *SDH2* with the inducible promoter of 3-ketoacyl-CoA thiolase encoding gene (*POT1*) from *Y. lipolytica*, which is almost inactive in *Y. lipolytica* grown on glucose or glycerol (Jost et al., 2014). When strain H222-AZ2 was fermented in controlled bioreactors with oxygen limitation, succinic acid was produced at 25 g/L titer and 0.26 g/g glycerol yield with 0.152 g/L/h productivity.

A different approach was undertaken by the research group of Qingsheng Qi, who deleted *SDH5* gene in PO1f strain (derivative of W29), obtaining PGC01003. This strain produced 198.2 g/L succinate on complex medium with glycerol in an *in situ* fibrous bed bioreactor (*is*FBB) with pH controlled at 6.0 (Li et al., 2017, 2018b). The yield was calculated at 0.42 g/g glycerol, however for complex media the yield numbers can be misleading because other media components are used as carbon sources as well. We will further only discuss the yields measured on mineral media with a single carbon source. The strain PGC01003 also had an impaired glucose metabolism, consuming <6 g/L glucose after 120 h in shake flask (Gao et al., 2016). The strain underwent adaptive laboratory evolution on glucose for 21 days to result in strain PSA02004, which produced 65.7 g/L succinate in complex medium with glucose at pH 6.0 (Yang et al., 2017). The strain was further evolved in an *is*FBB for around 60 days to give strain PSA3.0, which produced 18.4 g/L succinic acid at pH 3.0 (Li et al., 2018a). The authors also tried a rational approach, where the Δ*sdh5* strain PGC01003 was further engineered by deleting acetyl-CoA hydrolase (*ACH1*) and expressing phosphoenolpyruvate carboxykinase (*PCK*) from *S. cerevisiae* and endogenous succinyl-CoA synthase beta-subunit (*SCS2*) (Cui et al., 2017). The resulting strain PGC202 produced 110.7 g/L succinic acid on complex medium with glycerol without pH control (Cui et al., 2017); the authors did not report whether the ability to utilize glucose was restored in this strain. A year later, Yu et al. reported that the strain PGC202 was able to produce 53.6 g/L succinate in complex medium in bioreactors (Yu et al., 2018). However, in this study, the pH of the fermentation was not controlled and it dropped from 6.5 to only 5.1, which is contradictory to the theoretical pH drop that would occur at the concentration of succinic acid above 50 g/L (the final pH should be below 3, see also final pH values in fermentation without pH control in Table 1). The comparison of final succinic acid titer by all the developed strain so far (Table 1) shows that even the highest producer from glucose (about 50 g/L) is still far below the acceptable range of titer and productivity required for implementing in large-scale production. For commercial production of bulk chemicals using fermentation, the titers above 100–150 g/L are typically required and hence all the *Y. lipolytica* strains reported in literature so far are still far away from this goal.

In the current study, we engineered *Y. lipolytica* to produce succinic acid in mineral medium with glucose as the sole carbon source. The main aim was to construct the strain through rational metabolic engineering approach, rather than mutagenesis, to have the final strain fully genetically defined. The strain was engineered rationally by downregulating the expression of *SDH1*, which unlike *SDH* deletion, had no inhibitory effect on cell growth on glucose. The succinic acid production in this strain was further improved by optimizing the flux toward succinate through manipulation of the glyoxylate pathway, oxidative TCA branch, and reductive carboxylation pathways. The strain carries nine specific genome edits and presents a platform for further rational engineering toward succinic acid production.

## Materials and Methods

### Strains, Culture Conditions, and Chemicals

*Escherichia coli* strain DH5α was used for cloning and plasmid propagation. Lysogeny Broth (LB) liquid medium or LB solid medium supplemented with 20 g/L agar containing 100 mg/L ampicillin was used for *E.coli* cultivation at 37°C.

*Yarrowia lipolytica* ST6512 with the genotype ku70Δ cas9::DsdA MatA was used as the parental strain. To construct this strain, simultaneous ku70 disruption and Cas9 insertion were performed by transforming linearized pCfB6364 into *Y. lipolytica* W29. The plasmid bears dsdAMX selection marker (Stovicek et al., 2015), which enables growth on D-serine as the sole nitrogen source (Vorachek-Warren and McCusker, 2004). All the strains are listed in Supplementary Table 1. The media used for growth of *Y. lipolytica* strains contained 10 g/L yeast extract, 20 g/L peptone (YP media), supplemented with 5% v/v glycerol (YPG media) or 2% w/v D-glucose monohydrate (YPD media), unless other concentrations are stated. Antibiotics were also supplemented when needed at following concentrations: hygromycin B (Thermo Fisher Scientific) at 400 mg/L and nourseothricin (Jena Bioscience GmbH) at 250 mg/L. The chemicals were all obtained from Sigma-Aldrich, unless otherwise mentioned.

### Plasmid Construction

All plasmids and BioBricks used are listed in Supplementary Tables 2, 3, respectively. BioBricks were amplified by PCR using Phusion U polymerase (Thermo Fisher Scientific) with following thermal program: 98°C for 30 s, 30 cycles of (98°C for 10 s, 51°C for 30 s, 72°C for 30 s/kb), and 72°C for 5 min. BioBricks were then resolved on 1% agarose gels and purified using NucleoSpin^®^Gel and PCR Clean-up kit (Macherey-Nagel). The assembly of BioBricks into vectors was conducted by USER cloning (Holkenbrink et al., 2017). The parental vectors were digested with FastDigest SfaAl (Thermo Fisher Scientific) at 37°C for 60 min and nicked with Nb.BsmI (New England BioLabs) at 65°C for 60 min. BioBricks with compatible overhangs and SfaAl/Nb.BsmI-treated parental vectors prepared as above were incubated in CutSmart^®^ buffer with USER enzyme (New England BioLabs) for 25 min at 37°C, followed by 10 min at 25°C, and transformation into *E. coli*. The colonies were tested by colony PCR and correct assembly was verified by DNA sequencing.

### Construction of *Y. lipolytica* Strains

All the integrative vectors together with gRNA vectors are listed in Supplementary Table 2. Plasmids and BioBricks were transformed into parental strains by standard lithium acetate protocol (Chen et al., 1997) described in detail elsewhere (Holkenbrink et al., 2017). All the integration vectors were linearized with endonuclease NotI (Thermo Fisher Scientific) and gel purified for yeast transformation.

Genome editing of the strains, including gene knockout, and integration of single or multiple genes, were all performed according to the EasyCloneYALI Toolbox (Holkenbrink et al., 2017). Briefly, strain ST8218 with deleted gene *YALI0E30965g* (*ACH1*) was constructed by transforming 500–1,000 ng gRNA helper vector together with 500–1,000 ng of the repair template, which encodes 500 up- and 500 bp downstream sequences around the *ACH1* open reading frame. The required repair templates were made according to EasyCloneYALI Toolbox (Holkenbrink et al., 2017). The right colonies were selected by colony PCR using OneTaq Master Mix (New England Biolabs) using the sequencing primers listed in Supplementary Table 4. For strain ST8507 with down-regulated *SDH1* expression, crRNA sequence of gRNA vector was found by identifying the promoter region of gene *YALI0D11374g* (*SDH1*) as the target site on the online tool “CHOPCHOP” (http://chopchop.cbu.uib.no).

Screening of *Y. lipolytica* strains was carried out in 24-deep-well plates with air-penetrable metal lids (EnzyScreen, The Netherlands) containing 3 mL of YPG media with 60 g/L glycerol. A pre-culture of each *Y. lipolytica* strain was grown in 5 mL YPG medium (60 g/L glycerol) in 13-mL tubes overnight at 30°C and 250 rpm. The inoculation of all strains was done with an adequate amount of pre-culture to obtain the starting OD_600_ = 0.1. The plates were incubated at 30°C with shaking at 300 rpm at 5 cm orbit cast. Samples were taken after 4 days and analyzed for cell growth and succinic acid titer. All the experiments were run in triplicates, the average and standard deviation values are reported. All the measured standard deviations were below 20%.

### Shake Flask Fermentation

The shake flask cultivations were carried out in either 250 or 500 mL shake flasks containing YPG or YPD media at the volume of 10 or 20% of the total flask volume. The flasks were inoculated with overnight pre-culture (described in section 0) to a starting OD_600_ of 0.1, and incubated at 30°C with shaking at 250 rpm. In the case of fermentation at neutral pH, solid CaCO_3_ corresponding to final concentration of 10 g/L was sterilized together with the shake flask, and the sterile medium was then added aseptically. Samples of 200 μL were taken aseptically at specific time intervals. All the experiments were run in triplicates, the average and standard deviation values are reported. All the measured standard deviations were below 20%.

### Adaptation on Glucose

Adaptation of succinic acid producing *Y. lipolytica* strain on glucose was conducted in 250 mL shake flasks containing 50 mL of YPD medium with subsequently increasing initial glucose concentration. The adaptation was carried out in 3 independent lines and the transfer to new media was performed separately for each line. In first step, named as “transfer 1,” the shake flasks containing YPD with 20 g/L glucose were inoculated from overnight growing cells in YPG with 60 g/L glycerol to get an OD_600_ = 0.1. The cells were then grown at 30°C and 250 rpm up to OD_600_ 5, after which an adequate amount was transferred to new medium to get an initial OD_600_ = 0.1. This process was repeated with increasing glucose concentrations in the medium.

To separate and purify the adapted isolates, the exponentially growing cells of the final transfer were serially diluted and cultivated on YPD plates containing 20 g/L glucose for 72 h at 30°C. Single isolates were tested for succinic acid production.

### Fed-Batch Fermentation in Bioreactors

Fed-batch fermentation for succinate production was carried out in 1 L bioreactors (BIOSTAT^®^ Q plus, Sartorius, Goettingen, Germany). The bioreactors were equipped with measurement probes for pH, dissolved oxygen (DO), temperature and cell density. During the fermentation, off-gas CO_2_ and O_2_ concentration was monitored continuously (Thermo Scientific Prima BT MS). Data acquisition was achieved with Lucullus software (Securecell AG, Switzerland). The seed culture was prepared by cultivating the adapted strain in 100 mL of mineral medium described by Maury et al. (2018) containing 20 g/L glucose, and incubating overnight at 30°C and 250 rpm. The seed culture was then centrifuged, washed twice and resuspended in 10 mL media and inoculated to the bioreactors containing 400 mL mineral medium (Maury et al., 2018) to a starting OD_600_ of 1.0. Fermentation was carried out at 30°C, with 1 vvm aeration and pH controlled at 5.0 by addition of 2M KOH. During the cultivation, the dissolved oxygen (DO) level was kept above 40% by cascaded control of stirrer speed and air input flow rate. For repeated fed-batch, sterile glucose was pulsed to a final concentration of 25–50 g/L in the reactor after observing a significant drop in CO_2_ levels.

### Analytical Methods

In shake flask and deep well-plate experiments, cell growth was monitored by measuring the optical density at 600 nm (OD_600_) using NanoPhotometer (Implen GmbH, Germany). In the case of buffered fermentation, the samples were diluted with 1 M HCl to dissolve carbonate salts. To conversion of OD_600_ values to dry cell weight (DCW) was done by following empirical correlation:

$$\begin{array}{r} DCW\left(g/L\right)=0.1394\times OD_{600}-0.0029 \end{array}$$

The activity of *SDH* enzyme in the strains was measured by using calorimetric assay kit (Sigma Aldrich, Denmark). Cell-free samples were prepared by homogenizing 1 × 10^6^ cells in 100 μL of ice cold *SDH* Assay Buffer (provided by manufacturer) and centrifuging at 10,000 × *g* for 5 min. *SDH* activity was determined by generating dichlorophenolindophenol (DCIP) as product with absorbance at 600 nm, which is proportional to the enzymatic activity present. One unit of succinate dehydrogenase was defined as the amount of enzyme that generated 1.0 μmole of dichlorophenolindophenol (DCIP) per minute at pH 7.2 at 25°C.

Glucose, glycerol, succinic acid, and mannitol were all detected and quantified using high performance liquid chromatography (HPLC) Agilent 1,100 series with a refractive index detector and a Bio-Rad Aminex HPX-87H column (300 × 7.8 mm) with 5 mM H_2_SO_4_ as an eluent at a flow rate of 0.6 mL/min with column oven temperature set to 30°C. To protect the HPX-87H column from contamination and foreign particles, a guard column was fitted to the system. In repeated fed-batch fermentations in bioreactor, glucose concentration of the samples was measured enzymatically by YSI 2900 Biochemistry Analyzer (USA). The yield of succinic acid was defined as the amount of final succinic acid/succinate produced from 1 g of carbon source consumed.

## Results and Discussion

### Metabolic Engineering of *Y. lipolytica* for Succinate Production

The cells synthesize succinate as an intermediate of the TCA cycle in mitochondria and as a product of glyoxylate cycle in the cytosol and peroxisomes. Succinate produced in glyoxylate pathway is transported into mitochondria, where it enters the TCA cycle. If succinate dehydrogenase *SDH* that converts succinate to fumarate were inactivated, then TCA and glycoxylate pathways would function in the linear mode with succinate as the product. However, succinate dehydrogenase is an essential enzyme in the obligatory aerobic *Y. lipolytica* and this enzymatic activity cannot be completely eliminated, but only attenuated. Hence, our strategy was to reduce the activity of *SDH* by decreasing gene expression.

To ease the strain engineering, we deleted *KU70* gene that participates in non-homologous end-joining and introduced *Y. lipolytica* codon-optimized *cas9* gene from *Streptococcus pyogenes* into the genome of *Y. lipolytica* strain W29. The resulting strain ST6512 had only a slightly lower maximum specific growth rate (0.43 ± 0.04 h^−1^) in comparison to the parental W29 strain (0.46 ± 0.03 h^−1^) (Supplementary Figure 1).

Further strain metabolic engineering was to interrupt TCA cycle, by trying to knock out the *SDH5* gene, however this was not possible to do in our strain. In parallel, we continued with inactivation of the mitochondrial acetate production in ST6512, which would occur upon interruption of the TCA cycle and would cause growth inhibition (Gao et al., 2016; Cui et al., 2017). For this, we deleted *YALI0E30965g* gene, encoding the mitochondrial acetyl-CoA hydrolase (*ACH1*). The resulting strain (ST8218) and all the derived strains produced no acetate in fermentation experiments. Next step was the introduction of dicarboxylic acid transporter *SpMae1* from *Schizosaccharomyces pombe* in order to improve the secretion of succinic acid. This transporter has been proven to increase malic acid titer significantly in *S. cerevisiae* (Zelle et al., 2008). We also recently showed that *SpMae1* transporter is likely a *SLAC1* voltage-dependent anion channel and is thus very energetically efficient (Darbani et al., 2019). Hence, we expressed transporter gene *SpMae1* in Δ*ACH1* ST8218, to obtain strain ST8413.

Then we proceeded with downregulating the activity of succinate dehydrogenase, as an alternative method for *SDH* deletion. The enzyme consists of five protein subunits, each encoded by a separate gene (*SDH1* to *SDH5*). *SDH5* has been reported to be knocked out in *Y. lipolytica* W29 strain before (Gao et al., 2016). However, all our attempts to knock out this gene in the same background strain W29 with four different designed crRNAs (assembled to plasmids pCfB8032, pCfB8033, pCfB8114, and pCfB8115, Supplementary Table 2) were unsuccessful. We also tried to delete this gene using dominant selection marker (hygromycin B resistance) and large 500 bp homologous arms. This method typically works very well in *Y. lipolytica*, but we still could not obtain any colonies for *SDH5* deletion mutants on either glycerol- or glucose-based media. This indicates that *SDH5* is an essential gene. Two other subunits of succinate dehydrogenase *SDH1* and *SDH2* were reported to be essential previously (Yuzbashev et al., 2010). Hence, we decided to downregulate the *SDH1* gene like following. The endogenous promoter of *SDH1* gene was truncated to 95 bp upstream of the start codon (*t*P*SDH1*) in strain ST8413. The resulting strain ST8507 accumulated 1.2 ± 0.1 g/L succinate in 4 days in deep-well plate cultivation, while no succinate was produced by the parental strain ST8413 (Figure 2). The comparison between *SDH* activity in the parental strain ST8413 (0.012 ± 0.001 milliunits/μL) and in *SDH1* promoter truncation strain ST8507 (0.003 ± 0.000 milliunits/μL) confirmed a significant reduction of *SDH* activity by 77% (Supplementary Figure 1). Further, the strain ST8507 had a lower growth with μ_max_ decreasing from 0.49 ± 0.01 h^−1^ to 0.33 ± 0.02 h^−1^ and an 8-h longer lag phase (Supplementary Figure 2). This indicates the significant role of this gene in the cell growth and metabolism.

**Figure 1:**
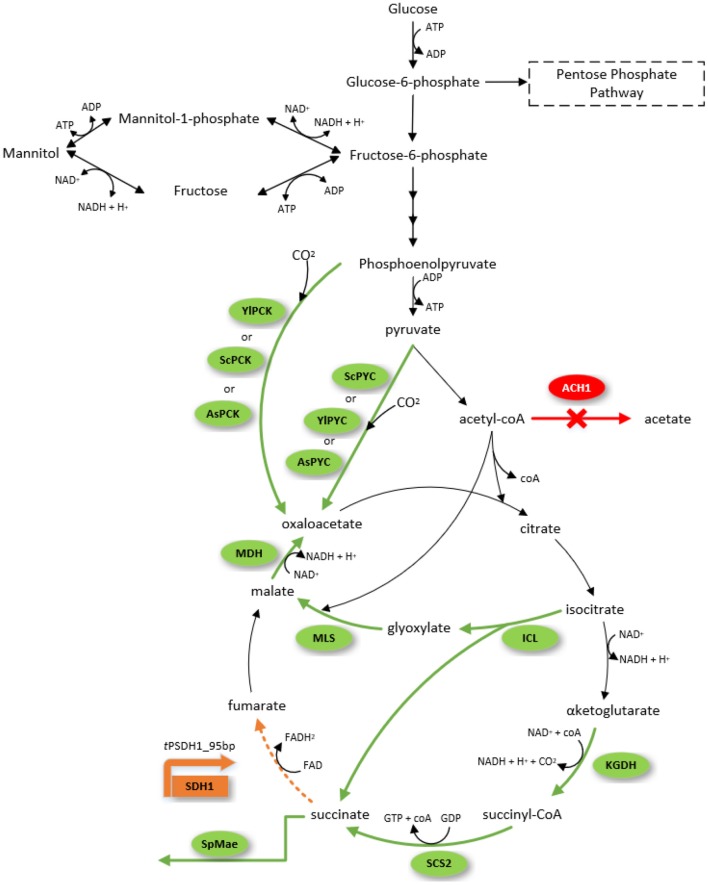
Succinic acid and mannitol biosynthesis pathway from glucose as the sole carbon source in *Yarrowia lipolytica*. The engineered route for overexpression of genes in this study is highlighted with green arrows, deleted pathways highlighted in red, and downregulated pathway in orange. Gene nomenclature: Acetyl-CoA hydrolase (*ACH1*), phosphoenolpyruvate carboxykinase from *A. succinogenes* (*AsPCK*), pyruvate carboxylase from *A. succinogenes* (*AsPYC*), Isocitrate lyase (*ICL*), α-ketoglutarate dehydrogenase (*KGDH*), malate dehydrogenase (*MDH*), malate synthase (*MLS*), pyruvate carboxylase from *S. cerevisiae* (*ScPYC*), phosphoenolpyruvate carboxykinase from *S. cerevisiae* (*ScPCK*), succinyl-CoA synthase beta-subunit (*SCS2*), dicarboxylic acid transporter from *S. pombe* (*SpMae1*), pyruvate carboxylase from *Y.lipolytica* (*YlPYC*), phosphoenolpyruvate carboxykinase (*YlPCK*), succinate dehydrogenase subunit 1 under the control of truncated promoter (*tPSDH1*).

**Figure 2:**
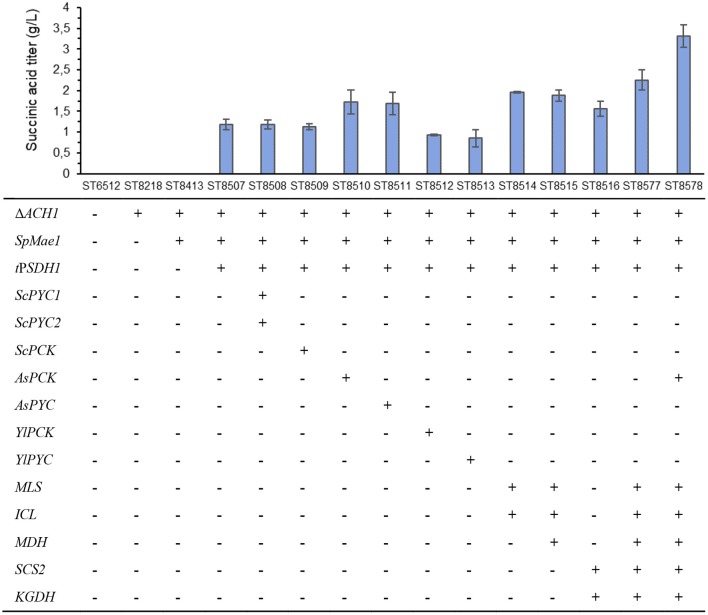
Succinic acid production by engineered strains of *Y. lipolytica*. The strains were cultivated on YP medium with 5% v/v of glycerol as carbon source in deep-well plates for 4 days. The highest producing strain ST8578 was selected for further study. The values are averages from three biological replicates, the error bars show the standard deviation.

The strain was further engineered to improve succinic acid production through three different strategies (Figure 1). The first strategy was to enhance the carboxylation of reactions that generate oxaloacetate for the TCA cycle. Here, we expressed pyruvate carboxylase encoding genes: native (*PYC*), from *S. cerevisiae* (*ScPYC1* and *ScPYC2*) or from *A. succinogenes* (*AsPYC*). We also tested the expression of phosphoenolpyruvate carboxykinase gene: native (*PCK*), from *S. cerevisiae* (*ScPCK*), or from *A. succinogenes* (*AsPCK*), to investigate whether *PYC* or *PCK* pathways were more effective on improving succinic acid titer. The second strategy was directed to increasing the flux through glyoxylate pathway, where additional copies of the native isocitrate lyase (*ICL*), malate synthase (*MLS*), and malate dehydrogenase (*MDH*) genes were expressed from strong constitutive promoters. The third strategy aimed to increase the flux through the oxidative TCA branch. For this, we expressed additional copies of succinyl-CoA synthase beta subunit (*SCS2*) and α-ketoglutarate dehydrogenase (*KGDH*) genes, again driven by strong constitutive promoters. A total of 11 strains were constructed and tested for succinate production on complex medium with glycerol as carbon source in deep-well plates (Figure 2).

For the carboxylation strategy, we saw a 42 and 45% improvement of succinate titer upon expression of pyruvate carboxylase and PEP carboxykinase from *A. succinogenes*, respectively (Figure 2). The expression of *Yarrowia* native or *S. cerevisiae* enzyme variants did not improve succinate production.

The glyoxylate pathway strategy, where *ICL, MLS*, and *MDH* genes were overexpressed resulted in 58% improvement of the succinate titer. The native glyoxylate pathway genes are repressed during the growth on glucose (Juretzek et al., 2001), while we used strong constitutive TEF promoter with its intron (pTEFin) for *ICL* expression (Tai and Stephanopoulos, 2013) and thus activated the pathway. These results can be compared with the results obtained by Cui et al., who expressed *ICL* gene under the control of *HP4D* promoter and could not see any improvement in succinate titer (Cui et al., 2017). In this study, we overexperessed *ICL* gene from *TEF1* promoter with intron and we observed a positive effect on succinic acid production. The TEF1 promoter was shown to be 5-fold stronger than *HP4D* promoter in *Y. lipolytica* (Tai and Stephanopoulos, 2013).

Overexpression of the oxidative branch of the TCA cycle genes (*SCS2* and *KDGH*) alone did not give an improvement of succinate production, but when combined with glyoxylate strategy, an improvement of 90% was obtained in comparison to the reference strain ST8507. These results shows the synergic and additive effect of genes in glyoxylate shunt and oxidative branch of TCA cycle, on succinic acid titer. Finally, we combined all three strategies (from the first strategy, we chose *AsPCK* expression, as both *AsPCK* and *AsPYC* showed similar effect on succinic acid titer) into strain ST8578 that gave 3.3 ± 0.2 g/L succinic acid, a 280% increase in comparison to the reference ST8507.

### Growth and Succinic Acid Production of the Engineered Strain

**Figure 3:**
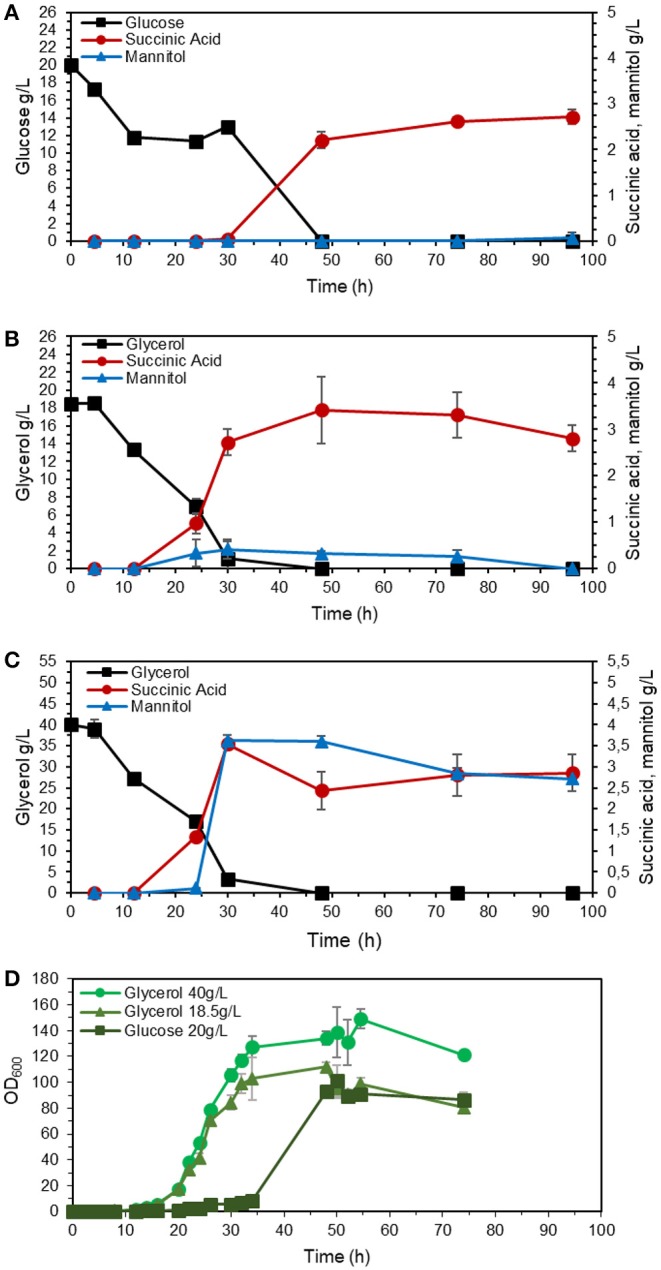
Performance of succinate-producing strain (ST8578) on glucose or glycerol as carbon source. The strains were cultivated in shake flasks on YP medium with 20 g/L glucose monohydrate **(A)**, 18.5 g/L glycerol **(B)**, or 40 g/L glycerol **(C)**, with OD_600_ profile showing the ability of the cells to grow on glucose with longer lag phase compared to glycerol **(D)**. The values are averages from three biological replicates, the error bars show the standard deviation.

The strains with inactivated *SDH5* were previously reported to lose the ability to grow on glucose (Yang et al., 2017). To investigate the effect of reduced expression of *SDH1*, we studied the growth and product formation profile of the engineered strain ST8578 on complex medium, as shown in Figure 3, with glucose or glycerol as carbon source (0.6 C-mol substrate/L). The strain grew on glucose medium, however, the lag phase was longer compared to growth on glycerol (16 vs. 6 h). There were also distinct differences between the metabolites production on two media. Succinic acid was produced at a higher titer in glycerol medium compared to glucose medium, 3.4 ± 0.7 vs. 2.2 ± 0.1 g/L and with a higher yield, 0.20 ± 0.04 C-mol/C-mol glycerol vs. 0.13 ± 0.01 C-mol/C-mol glucose (Table 2). While succinic acid titer was higher on glycerol, there was also a by-product mannitol produced at 0.30 ± 0.04 g/L mannitol, which was not detected in glucose fermentation. When we used higher starting glycerol concentration, succinic acid titer decreased slightly, while mannitol increased to 3.6 ± 0.1 g/L. This presents a problem for using glycerol as carbon source for succinic acid production, because a large fraction of carbon is channeled to mannitol.

**Table 2:** Shake flask fermentation results of ST8578 on YP media supplemented with 1.3 C-mol/L glycerol (40 g/L), 0.6 C-mol/L glycerol (18.5 g/L), and 0.6 C-mol/L D-glucose (20 g/L) after 48 h of cultivation.

**Parameter**	**Glucose 0.6 C-mol/L**	**Glycerol 0.6 C-mol/L**	**Glycerol 1.3 C-mol/L**
Succinic Acid titer (g/L)	2.2 ± 0.1	3.4 ± 0.7	2.8 ± 0.4
Yield (C-mol_SA_/C-mol_substrate_)	0.13 ± 0.01	0.20 ± 0.04	0.07 ± 0.01
Mannitol titer (g/L)	0	0.3 ± 0.0	3.6 ± 0.1

Glycerol is a more reduced substrate than glucose and generates an additional mol of NADH during the catabolism. If the culture is oxygen limited (as may happen at higher cell densities), the rate of NAD^+^ regeneration via oxidative phosphorylation will be slower than NADH generation (Diano et al., 2006). To manage the redox co-factor imbalance, *Y. lipolytica* begins synthesizing mannitol regenerating NAD^+^ in the process (Workman et al., 2013). Additionally, polyols as mannitol function as osmoprotectants, and their synthesis may be further induced by exposure to high concentrations of the substrate.

### Adaptation of Final Engineered Strain on Glucose

While the strain engineered through downregulation of *SDH1* preserved the ability to metabolize glucose, it had a longer lag phase on glucose than on glycerol and grew slower. We then carried out a short adaptation to shorten the lag phase and to increase the tolerance of the cells to high glucose concentrations.

**Figure 4:**
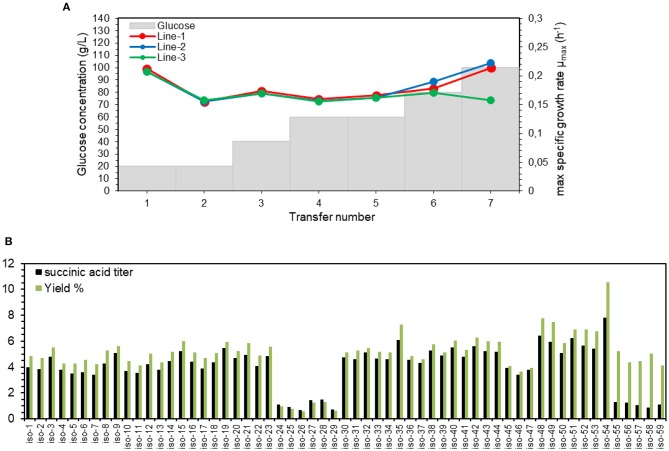
Adaptation of succinate-producing strain ST8578 to high concentrations of glucose. **(A)** Maximum specific growth rates (μ_max_) are shown as the strains are serially transferred to media with higher glucose concentrations. **(B)** Succinate titer and yield of multiple adapted isolates of ST8578 was assessed in deep-well plates on YP medium with 100 g/L glucose after 48 h incubation **(B)**. The isolate nr. 54 (iso-54) was chosen as the best producer.

For this purpose, a series of experiments in shake flasks with YP media containing initially 20 g/L glucose was started in 3 separate lines. The cells were transferred to the new medium when OD_600_ reached 5. If this OD value was reached within <20 h, then a serial transfer was done into a medium with a higher glucose concentration, otherwise glucose concentration in the next transfer was kept the same. The initial decrease in μ_max_ from transfer number 1–2 is due to the presence of glycerol in the 1st transfer, which was inoculated with YPG-grown preculture (Figure 4A). The average μ_max_ of all the three lines of transfers 2–5 was 0.16 ± 0.00 h^−1^, which is close to that on YPD with 20 g/L glucose (Table 2). This implies that with 5 transfers, the cells were adapted to utilize 60 g/L of glucose with no change in the maximum specific growth rate. For transfers 6 and 7 (80 and 100 g/L glucose), μ_max_ value increased for lines 1&2 to 0.21 h^−1^, while it decreased for line 3 to 0.15 h^−1^.

As the adapted culture likely is a mixed population of different clones, we isolated single clones. A total of 59 single colonies were isolated from transfer 7 of Figure 4B, where 23 colonies were from line 1 (iso-1 to 23), 18 colonies from line 2 (iso-24 to 41), and 18 colonies from line 3 (iso-42 to 59). The results of deep-well plate fermentation of these 59 isolates in the medium containing 100 g/L glucose (Figure 4B) showed that isolates 48–54 from the slower growing line 3 had the highest succinic acid titer. Also from Figure 4B, it can be seen that the isolates 24–29 had improved growth on glucose through adaptation process, but had lost succinic acid production ability during this time course. The highest titer of succinic acid was for isolate 54, with 7.8 ± 0.0 g/L succinic acid after 48 h, corresponding to 0.105 g/g glucose yield, which was also the highest among the isolates. Therefore, after only 7 transfers, the isolate 54 (named adapted-ST8578) was obtained that was able to grow on YPD containing 100 g/L glucose.

**Figure 5:**
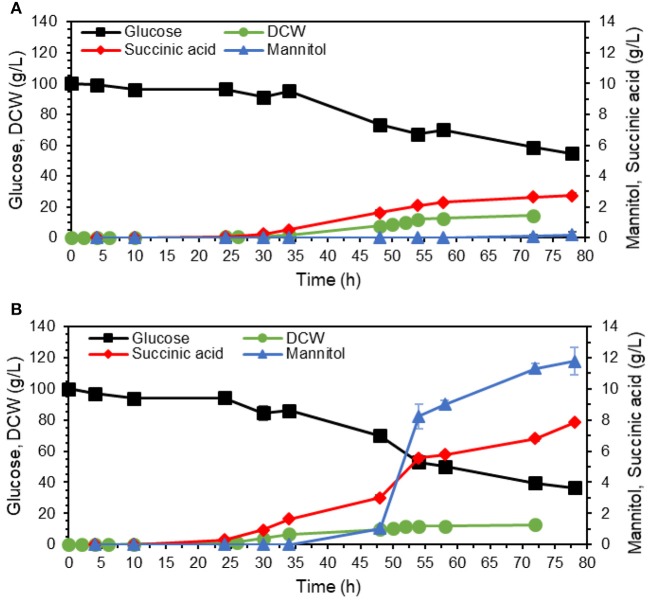
Comparison of the succinate-producing strain ST8578 **(A)** and its adapted isolate (iso-54) **(B)**. The strains were cultivated in shake flasks with YP medium containing 100 g/L glucose as carbon source. The values are averages from three biological replicates, the error bars show the standard deviation.

The adapted strain was compared to the parental strain in 250 mL shake flasks containing YPD with 100 g/L glucose. The adaptation has shortened the lag phase for ~6 h (Figure 5), with the final biomass concentrations of 14.5 ± 0.5 g DCW/L and 12.8 ± 0.1 g DCW/L for non-adapted and adapted strains, respectively. The μ_max_ increased from 0.14 ± 0.01 to 0.18 ± 0.00 h^−1^. The final titer of succinic acid increased 2.8-fold, from 2.7 ± 0.2 g/L succinic acid for the non-adapted strain to 7.8 ± 0.0 g/L for the adapted strain (Figure 5). Furthermore, the adapted strain utilized a higher fraction of the added glucose (63.6 ± 2.9 g/L of glucose vs. 45.5 ± 3.6 g/L of glucose for the non-adapted strain). Interestingly, the adapted strain produced large amount of mannitol, 11.8 ± 0.9 g/L vs. 0.2 ± 0.1 g/l for the non-adapted strain.

### Suppression of Mannitol Production in the Adapted Strain

**Figure 6:**
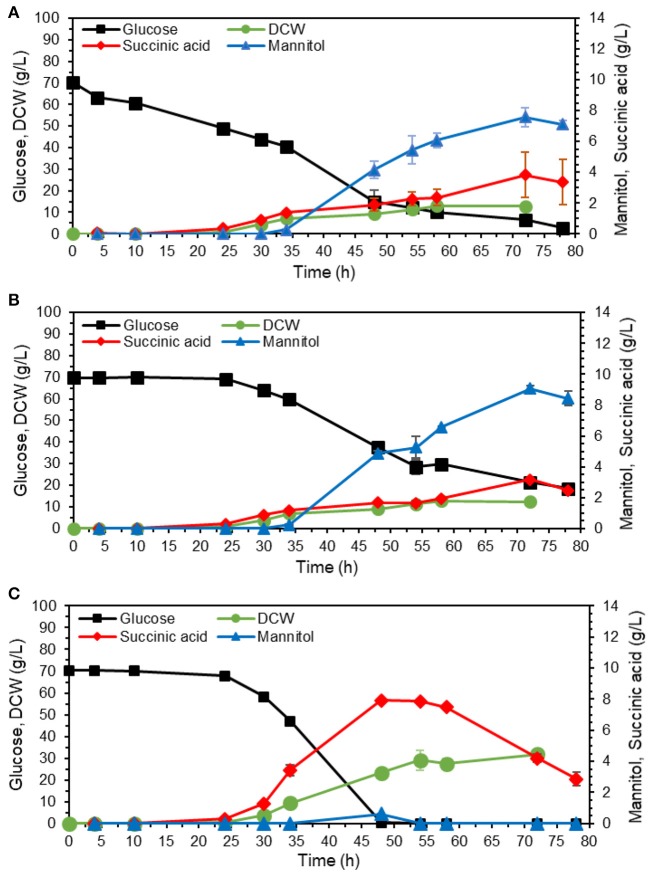
Effect of aeration rate and medium buffering on mannitol production. The adapted isolate 54 was cultivated in shake flasks on YP with 70 g/L glucose. **(A)** Cultivation volume was 20% of the total flask volume. **(B)** Cultivation volume was 10% of the total flask volume to obtain better aeration. **(C)** Cultivation volume was as in A, medium was buffered with 10 g/L calcium carbonate. The values are averages from three biological replicates, the error bars show the standard deviation.

Mannitol is a sugar alcohol that is involved in carbohydrate storage, redox co-factor balancing, osmoprotection, and oxidative stress protection (Patel and Williamson, 2016). The pathway, also shown in Figure 1, is known to be activated under oxygen limitation (Diano et al., 2006). We tested whether higher aeration would decrease mannitol production by filling up the flasks with medium up to 10% instead of 20% of the total volume (Figures 6A,B). Based on results from Figure 5, the adapted cells were cultivated in YPD media containing 70 g/L, corresponding to glucose amount that could be utilized by the strain in 80 h (Figure 5B).

Contrary to our hypothesis, the final mannitol titer was actually higher in the flask with better aeration (10% working volume), 8.4 ± 0.5 vs. 7.6 ± 0.6 g/L in the flask with normal aeration (20% working volume). The final biomass was similar for both conditions, 12.8 ± 1.1 g DCW/L for normal aeration (Figure 6A) and 12.4 ± 0.3 g DCW/L for high aeration (Figure 6B). These results imply that mannitol overproduction by the adapted strain was not caused by oxygen limitation. We then hypothesized that mannitol production could instead be caused by acidic pH stress. To investigate the effect of pH, we supplemented YPD medium containing 70 g/L glucose with 10 g/L CaCO_3_ for buffering (Figure 6C) and indeed this supplementation abolished mannitol production. Moreover, the glucose was depleted already at 48 h in contrast to 78 h in unbuffered fermentation. After glucose depletion, the cells started using succinate as the substrate. The final biomass at glucose depletion point was nearly double for the buffered fermentation, 23.3 ± 0.7 g DCW/L compared to 12.8 ± 1.1 g DCW/L. With no carbon wasted on mannitol synthesis, buffered fermentation also resulted in more succinate, 7.9 ± 0.1 g/L with a yield of 0.11 ± 0.00 g/g glucose (Figure 6C) compared to 3.3 ± 1.5 g/L and 0.05 ± 0.02 g/g glucose for unbuffered fermentation (Figure 6A). The secretion of mannitol started nearly at the same time as succinate production (Figures 5, 6), which confirms our hypothesis that mannitol production may be a cellular response to the acidic pH.

### Fed-Batch Fermentation in Bioreactors

**Figure 7:**
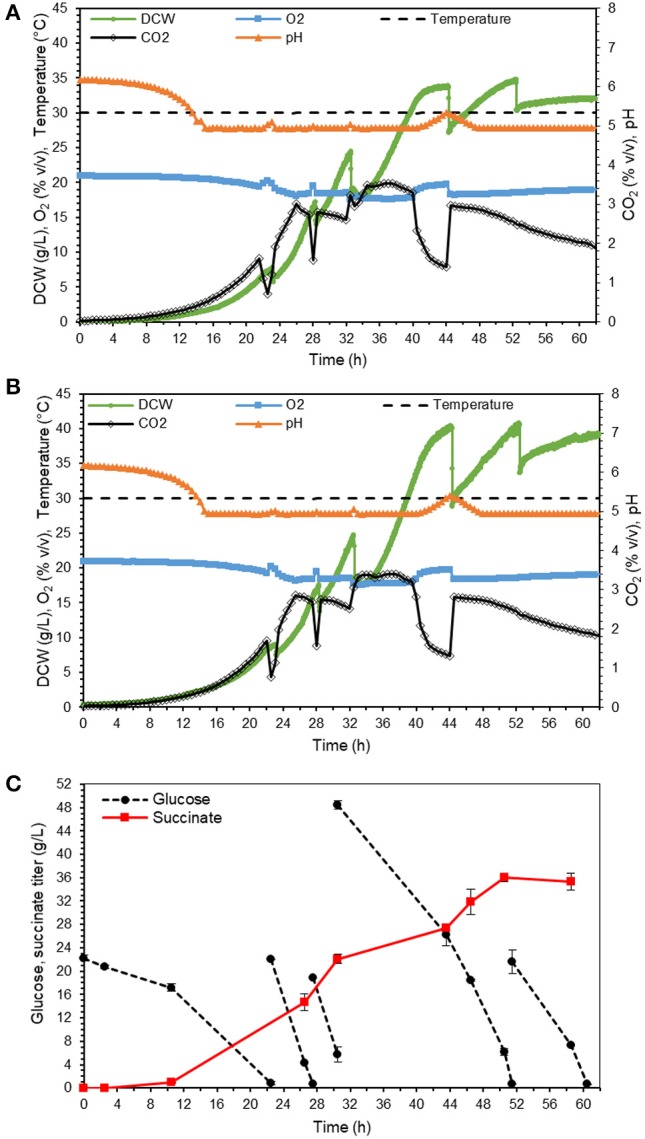
Bioreactor fermentation results of adapted strain in minimal medium in two 1 L fermenters, with online data for individual reactors **(A,B)**, and offline measurements as average for both reactors **(C)**, error bars indicate standard deviation calculated from the two independent bioreactor experiments.

Finally, we tested the production of succinic acid in mineral medium at pH 5 in two controlled repeated fed-batch mode operated bioreactors. At this pH, the major fraction of product is in acidic form rather than dissociated form (according to calculations based on K_a_ values of succinic acid 6.2 × 10^−5^ and 2.3 × 10^−6^), which means that no succinic acid recovery is needed in the downstream process. The feeding was aimed to provide 25–50 g/L glucose to the culture on the time points of glucose depletion, which was indicated by CO_2_ production drop or pH increase. The batch phase with exponential growth was completed at around 22 h, after which the first pulse of feeding was spiked. As shown in Figures 7A,B for individual bioreactors, the process was highly reproducible with 32 and 39 g DCW/L and final succinate titer of 35.3 ± 1.5 g/L in 59 h of fermentation (Figure 7C). The yield 0.26 ± 0.00 g/g glucose was almost double as high as in the shake flask cultivations (section Suppression of mannitol production in the adapted strain). The productivity was 0.60 ± 0.03 g/L/h. The maximum uptake rate of glucose for adapted strain was 1.0 ± 0.03 g/L.h during batch phase. With similar growth condition and biomass concentration of 11 g/L, Moeller et al. reported a maximum glucose uptake rate of 1.1 ± 0.14 g/L.h for *Y. lipolytica* (Moeller et al., 2012). Comparison of these two values shows that the adapted strain has almost recovered all its capability of glucose utilization. However, the glucose consumption rate of Y. lipolytica is still much lower than other yeast species. For instance, the glucose uptake rate for succinate producer *S. cerevisiae* strain AH22ura3 (Raab et al., 2010) is more than two times higher than that of *Y. lipolytica*.

The comparison of the results in our study with the titer of highest succinate producer of *Y. lipolytica* strain VKPM Y-3753 from glucose (Bondarenko et al., 2017, Table 1) shows that the later produces about 20 g/L higher than our strain. However, the titer for both processes is at least 100 g/L away from where it should be for commercial implementation. Hence a lot of future work is needed on both strains to bring them to the performance level required for production of a bulk chemical as succinic acid. The main advantage of adapted-ST8578 constructed in this study is that it has been developed through rational metabolic engineering approach and it is fully genetically defined. In contrast, VKPM Y-3753, strain was created by multiple rounds of mutagenesis and selection. The adapted-ST8578 can be further genetically modified or evolved/mutagenized following similar strategies as described by Bondarenko et al. and will likely reach higher performance. Eventually, the commercial process will need to be carried out at a lower pH, possibly even avoiding base addition altogether. For this, further strain and process optimizations are needed that go beyond the scope of this study.

## Conclusion

The main aim of the study was to construct a variant of *Y. lipolytica* yeast capable of producing succinic acid from glucose through rational engineering. The successful engineering strategies included overexpression of glyoxylate pathway, oxidative TCA cycle branch, and reductive carboxylation, as well as expression of dicarboxylic acid transporter from *S. pombe*. A short adaptation of this strain on glucose reduced the lag phase and improved the growth rate at high glucose concentrations. The production of mannitol as by-product could be eliminated by maintaining a neutral pH. In fed-batch fermentation at pH 5, the succinic acid titer was 35.3 ± 1.5 g/L with the yield on glucose of 0.26 ± 0.00 g/g and volumetric productivity of 0.60 ± 0.03 g/L/h. At pH 5.0, the major fraction of product is in acidic form rather than dissociated form (according to calculations based on K_a_ values of succinic acid 6.2 × 10^−5^ and 2.3 × 10^−6^). Hence the ultimate goal of producing succinic acid from glucose was accomplished in the current work.

## Data Availability Statement

All datasets generated for this study are included in the article/Supplementary Material.

## Author Contributions

MB, KR, and IB conceived the study and designed the experiments and analyzed the data. MB performed the experiments. The fed-batch fermentation was performed by MB and SS. MB, KR, MH, SE, AN, and IB wrote the manuscript. IB and IA secured the funding and supervised the project.

### Conflict of Interest

The authors declare that the research was conducted in the absence of any commercial or financial relationships that could be construed as a potential conflict of interest.
