# Supplementary material for: Engineering Oleaginous Yeast as the Host for Fermentative Succinic Acid Production From Glucose
Source: Front Bioeng Biotechnol. 2019 Nov 27;7:361. doi: 10.3389/fbioe.2019.00361 (PMC6892388; doi:10.3389/fbioe.2019.00361)
Supplement: Supplementary file 1 [file Table_1.DOCX]

**Supplementary Figure 1**: Succinate dehydrogenase enzyme activity assay in parental strain ST8413 (W29 Cas9-DsdA:: ku70∆ ΔACH1 SpMae), and subsequent strain after promoter truncation ST8507 (ST8413-*t*PSDH1_95bp). One unit of succinate dehydrogenase was defined as the amount of enzyme in 1 µL of 10^6^ cell suspension that generated 1.0 μmole of dichlorophenolindophenol (DCIP) per minute at pH 7.2 at 25 °C. The values are averages from three biological replicates, and the error bars show the standard deviation.

**B**

**A**

**Supplementary Figure 2**: A. growth profile of parental strain ST4842 (*Y. lipolytica* W29), parental strain with integrated cas9 protein ST6512 (W29 Cas9-DsdA:: ku70∆), the strain with deleted ACH1 and integrated SpMae ST8413 (ST6512 ΔACH1 SpMae), and finally after promoter truncation ST8507 (ST8413-*t*PSDH1_95bp), with calculated maximum growth rate in B. The cells were grown in YPD with 20 g/L glucose. Genomic integration of cas9 protein into *Y. lipolytica* has no adverse effect on growth rate, while promoter truncation dramatically affected growth profile of the cell. The values are averages from three biological replicates, and the error bars show the standard deviation.

**Supplementary Table 1**. List of engineered *Y. lipolytica* strains

| Strain ID | Description | Parental strain | Integrated plasmid | Ref |
| --- | --- | --- | --- | --- |
| ST1 | *S. cerevisiae* CEN.PK113-7D |  |  | Peter Kötter |
| ST3683 | *Y. lipolytica* GB20 |  |  | (Angerer et al., 2014) |
| ST4842 | *Y. lipolytica* W29 (ATCC 24060) |  |  | ARS, NRRL^1^, USA |
| ST6512 | W29 Cas9-DsdA:: ku70∆ | ST4842 | pCfB6364 | (Marella et al., 2019) |
| ST8218 | ST6512-Δach1 | ST6512 | pCfB8035 | This study |
| ST8413 | ST8218-SpMAE1 | ST8218 | pCfB8190 | This study |
| ST8507 | ST8413-*t*PSDH1_95bp | ST8413 | pCfB8269 | This study |
| ST8508 | ST8507-ScPYC1-ScPYC2 | ST8507 | pCfB8182 | This study |
| ST8509 | ST8507-ScPCK | ST8507 | pCfB8183 | This study |
| ST8510 | ST8507-AsPCK | ST8507 | pCfB8184 | This study |
| ST8511 | ST8507-AsPYC | ST8507 | pCfB8185 | This study |
| ST8512 | ST8507-YlPCK | ST8507 | pCfB8186 | This study |
| ST8513 | ST8507-YlPYC | ST8507 | pCfB8187 | This study |
| ST8514 | ST8507-MLS-ICL | ST8507 | pCfB8188 | This study |
| ST8515 | ST8507-MLS-MDH-ICL | ST8507 | pCfB8189 | This study |
| ST8516 | ST8507-SCS2-KGDH | ST8507 | pCfB8191 | This study |
| ST8577 | ST8516-MLS-MDH-ICL | ST8516 | pCfB8189 | This study |
| ST8578 | ST8516-MLS-MDH-ICL-AsPCK | ST8516 | pCfB8189, pCfB8184 | This study |

**^1^ARS, NRRL-** Agricultural Research Service Culture Collection, USA

**Supplementary Table 2**. List of plasmids

| Plasmid ID | Description | Parent vector | BioBricks | Ref |
| --- | --- | --- | --- | --- |
| **Basic Integrative Vectors** | | | | |
| pCfB368 | pESC-URA-PYC2 |  |  | (Borodina et al., 2015) |
| pCfB2189 | pX-3-loxP-KlLEU2syn |  |  | (Stovicek et al., 2015) |
| pCfB3405 | pORI1001-Nat-CEN1-USER |  |  | (Holkenbrink et al., 2017) |
| pCfB3531 | FragEpi2Vec2 (GeneString) |  |  | (Holkenbrink et al., 2017) |
| pCfB4354 | pΔYALI0E28534-Leu |  |  | (Holkenbrink et al., 2017) |
| pCfB4589 | pPrtRNA-Gly-gRNA_GFP-NatMx |  |  | (Holkenbrink et al., 2017) |
| pCfB4906 | IntB-Hyg-PrTef-Cas9_codon_op |  |  | (Holkenbrink et al., 2017) |
| pCfB5932 | pΔku70-dsdSyn |  |  | (Holkenbrink et al., 2017) |
| pCfB6371 | pIntC-3-TPex20-Tlip2 |  |  | (Holkenbrink et al., 2017) |
| pCfB6681 | pIntE-3-Tpex20-Tlip2 |  |  | (Holkenbrink et al., 2017) |
| pCfB6682 | pIntC-2-Tpex20-Tlip2 |  |  | (Holkenbrink et al., 2017) |
| pCfB6684 | pIntD-1-Tpex20-Tlip2 |  |  | (Holkenbrink et al., 2017) |
| **Integration Vectors** | | | | |
| pCfB6364 | pku70-Tpex20-PrTef-Cas9-Ttef-Lip2-DsdSyn |  | BB1135, BB1965, BB1917,BB1792, BB1800 | This study |
| pCfB8182 | pIntC-2-<-ScPYC1-PexP-PGPD-ScPYC2-> | pCfB6682 | BB2174, BB2821, BB2822 | This study |
| pCfB8183 | pIntC-2-PexP-ScPCK-> | pCfB6682 | BB1558, BB2870 | This study |
| pCfB8184 | pIntC-2-PexP-AsPCK-> | pCfB6682 | BB1558, BB2823 | This study |
| pCfB8185 | pIntC-2-PexP-AsPYC-> | pCfB6682 | BB1558, BB2871 | This study |
| pCfB8186 | pIntC-2-PexP-YlPCK-> | pCfB6682 | BB1558, BB2868 | This study |
| pCfB8187 | pIntC-2-PexP-YlPYC-> | pCfB6682 | BB1558, BB2869 | This study |
| pCfB8188 | pIntD-1-<-MLS-PexP-PTEFin-ICL-> | pCfB6684 | BB3250, BB3251 | This study |
| pCfB8189 | pIntD-1-<-MLS-PexP-PGPD-MDH->Tpot1-PTEFin-ICL-> | pCfB6684 | BB3250, BB3252, BB3253 | This study |
| pCfB8190 | pIntC_3-Pexp-SpMae-> | pCfB6371 | BB1558, BB2872 | This study |
| pCfB8191 | pIntE-3<-SCS2-Pexp-PGPD-KGDH-> | pCfB6681 | BB2174, BB2866, BB2867 | This study |
| pCfB8239 | knock-out vector for SDH5 with Hph marker |  | BB1135, BB2956, BB1138, BB2957 | This study |
| pCfB8269 | Repair for PSDH1 truncation to 95bp | - | BB1135, BB3109, BB1138, BB3111 | This study |
| **gRNA vectors** | | | | |
| pCfB6371 | pIntC-3-Tpex20-Tlip2 |  |  | (Holkenbrink et al., 2017) |
| pCfB6627 | pNat-YLgRNA2_IntC_2 |  |  | (Holkenbrink et al., 2017) |
| pCfB6630 | pNat-YLgRNA3_IntC_3 |  |  | (Holkenbrink et al., 2017) |
| pCfB6631 | pNat-YLgRNA2_IntD_1 |  |  | (Holkenbrink et al., 2017) |
| pCfB6637 | pNat-YLgRNA3_IntE_3 |  |  | (Holkenbrink et al., 2017) |
| pCfB6818 | pNat-YLgRNA2_IntC_2-gRNA2_IntD_1 |  |  | (Holkenbrink et al., 2017) |
| pCfB8032 | pgRNA1-Nat-SDH5 | pCfB3405 | BB2683 | This study |
| pCfB8033 | pgRNA2-Nat-SDH5 | pCfB3405 | BB2684 | This study |
| pCfB8035 | pgRNA2-Nat-ACH1 | pCfB3405 | BB2686 | This study |
| pCfB8114 | pgRNA3-Nat-SDH5 | pCfB3405 | BB2814 | This study |
| pCfB8115 | pgRNA4-Nat-SDH5 | pCfB3405 | BB2815 | This study |
| pCfB8276 | pgRNA2-Nat-PSDH1 | pCfB3405 | BB3114 | This study |

**Supplementary Table 3.** List of BioBricks

| BioBrick ID | Description | PCR template | Forward primer | Reverse primer | Ref |
| --- | --- | --- | --- | --- | --- |
| BB1135 | Easy Clone vector backbone (digested with AsiSI and nicked with Nb.BsmI) | pCfB2189 | PR-11110 | PR-11111 | (Holkenbrink et al., 2017) |
| BB1138 | loxP-PrExp1-HphSyn-Ttef-loxP | pCfB3531 | PR-10593 | PR-10594 | (Holkenbrink et al., 2017) |
| BB1558 | PrExp | gDNA *Y. lipolytica* ST6512 | PR-15521 | PR-15522 | (Holkenbrink et al., 2017) |
| BB1563 | PrYlGPD1_for fusion-> | gDNA *Y.lipolytica* ST3683 | PR-15529 | PR-15528 | (Kildegaard et al., 2017) |
| BB1621 | <-PrExp_for fusion | gDNA *Y. lipolytica* ST3683 | PR-15605 | PR-15606 | (Kildegaard et al., 2017) |
| BB1635 | PrtRNA-Gly | pCfB4589 | PR-10607 | PR-15788 | (Holkenbrink et al., 2017) |
| BB1636 | crRNA-TRPR | pCfB4589 | PR-15789 | PR-10604 | (Holkenbrink et al., 2017) |
| BB1792 | ku70_down | gDNA *Y. lipolytica* ST4842 | PR-16708 | PR-16709 | This study |
| BB1797 | PrGPD | pCfB4354 | PR-141 | PR-16713 | This study |
| BB1798 | DsdAsyn | Gene String DNA fragment | PR-16714 | PR-16715 | (Stovicek et al., 2015) |
| BB1799 | TLip2 | pCfB4354 | PR-16716 | PR-142 | This study |
| BB1800 | loxP-PrGPD-DsdAsyn-Tlip-loxP | BB1797, BB1798, BB1799 | PR-141 | PR-142 | This study |
| BB1917 | Tpex20-PrTef-Cas9-Ttef-Tlip2 | pCfB4906 | PR-14148 | PR-14149 | (Holkenbrink et al., 2017) |
| BB1965 | ku70_up (int vector) | pCfB5932 | PR-16706 | PR-17826 | This study |
| BB2174 | <-PrEXP-PrGPD-> | BB1621, BB1563 | PR-15606 | PR-15528 | This study |
| BB2211 | PrTEFin_forfusion-> | gDNA *Y. lipolytica* ST3683 | PR-18931 | PR-18214 | This study |
| BB2660 | KO_ACH1_UP | gDNA *Y. lipolytica* ST6512 | PR-21809 | PR-21810 | This study |
| BB2661 | KO_ACH1_DW | gDNA *Y. lipolytica* ST6512 | PR-21811 | PR-21812 | This study |
| BB2683 | gRNA1_SDH5_cass1 | BB1635, PR-21805, PR-21806, BB1636 | PR-10607 | PR-10604 | This study |
| BB2684 | gRNA2_SDH5_cass1 | BB1635, PR-21807, PR-21808, BB1636 | PR-10607 | PR-10604 | This study |
| BB2686 | gRNA2_ACH1_cass1 | BB1635, PR-21815, PR-21816, BB1636 | PR-10607 | PR-10604 | This study |
| BB2687 | KO_SDH5_repair_template |  |  |  |  |
| BB2688 | KO_ACH1_repair_template | BB2660, BB2661 | PR-21809 | PR-21812 | This study |
| BB2731 | TPOT1->_forfusion | gDNA *Y. lipolytica* ST3683 | PR-21915 | PR-21916 | This study |
| BB2813 | <-PrExp-PrTEFin-> | BB1621, BB2211 | PR-15606 | PR-18214 | This study |
| BB2814 | gRNA3_SDH5_cass1 | BB1635, PR-21845, PR-21846, BB1636 | PR-10607 | PR-10604 | This study |
| BB2815 | gRNA4_SDH5_cass1 | BB1635, PR-21847, PR-21848, BB1636 | PR-10607 | PR-10604 | This study |
| BB2818 | ICL_forfusionPrTEfin-> | gDNA *Y. lipolytica* ST6512 | PR-22053 | PR-22054 | This study |
| BB2819 | MLS<- | gDNA *Y. lipolytica* ST6512 | PR-22055 | PR-22056 | This study |
| BB2820 | MDH-> | gDNA *Y. lipolytica* ST6512 | PR-22057 | PR-22058 | This study |
| BB2821 | ScPYC1<- | gDNA of *S. cerevisiae* ST1 | PR-22059 | PR-671 | This study |
| BB2822 | scPYC2-> | pCfB368 | PR-22060 | PR-673 | This study |
| BB2823 | AsPCK-> | synthetic Gene | PR-22061 | PR-7016 | This study |
| BB2866 | SCS2<- | gDNA *Y. lipolytica* ST6512 | PR-22337 | PR-22338 | This study |
| BB2867 | KGDH-> | gDNA *Y. lipolytica* ST6512 | PR-22339 | PR-22340 | This study |
| BB2868 | YlPCK-> | gDNA *Y. lipolytica* ST6512 | PR-22341 | PR-22342 | This study |
| BB2869 | YlPYC-> | gDNA *Y. lipolytica* ST6512 | PR-22343 | PR-22344 | This study |
| BB2870 | ScPCK-> | gDNA of *S. cerevisiae* ST1 | PR-22345 | PR-22346 | This study |
| BB2871 | AsPYC-> | pCfB8163 | PR-22347 | PR-22348 | This study |
| BB2872 | SpMae-> | pCfB2861 | PR-22365 | PR-7030 | This study |
| BB2956 | KO_SDH5_UP_U | gDNA *Y. lipolytica* ST6512 | PR-22390 | PR-22391 | This study |
| BB2957 | KO_SDH5_DW_U | gDNA *Y. lipolytica* ST6512 | PR-22392 | PR-22393 | This study |
| BB2993 | Tpot1::PrTEFin-> | BB2211,BB2731 | PR-21915 | PR-18214 | This study |
| BB3109 | KO_PSDH1_repair_UP | gDNA *Y. lipolytica* ST6512 | PR-22690 | PR-22691 | This study |
| BB3111 | KO_PSDH1_repair_gRNA2_Dw | gDNA *Y. lipolytica* ST6512 | PR-22694 | PR-22693 | This study |
| BB3114 | gRNA2_PSDH1 | BB1635, PR-22698, PR-22699, BB1636 | PR-10607 | PR-10604 | This study |
| BB3250 | <-MLS-PexP | BB2819, BB1621 | PR-15605 | PR-22056 | This study |
| BB3251 | PrTEFin-ICL-> | BB2211, BB2818 | PR-18931 | PR-22054 | This study |
| BB3252 | PGPD-MDH-> | BB1563, BB2820 | PR-15529 | PR-22058 | This study |
| BB3253 | Tpot1-PrTEFin-ICL-> | BB2731, BB2211, BB2818 | PR-21915 | PR-22054 | This study |

**Supplementary Table 4.** List of Primers. Underlined sequences represent overhangs used for USER cloning.

| Primer ID | Description | Sequence (5’ -> 3’) |
| --- | --- | --- |
| PR-10593 | Fragment2EpiVecYL _fw | agcaggctUGGAGGCGACGTGGCAG |
| PR-10594 | Fragment2EpiVecYL _rev | catgGCGGCCGCGAATGC |
| PR-10604 | tracrRNA_rev | CACGCGAUACCGTACCCACACAAAAAAAGCACCACCGACTC |
| PR-10607 | PrtRNAGly_fw | CGTGCGAUAGTGAATCATTGCTAACAGATC |
| PR-11110 | E.coli backboneUSER _fw | atcgcgtgcattcgcggccgcatttaaatcc |
| PR-11111 | E.coli backboneUSER _rev | atcgcacgcattcgcggccgcaaatttaaataaaatg |
| PR-141 | NB326URA3fwdU | agaacagcUgaagcttcgtacg |
| PR-14148 | TPex20-Tlip2_fw | acgtgcaacgctUacgcaactaacatgaatg |
| PR-14149 | TPex20-Tlip2_rev | agctgttcUcagatgcattcttgggcggtc |
| PR-142 | NB326URA3rev2U | AGGCCACUAGTGGATCTGATATCAC |
| PR-15521 | PrExp_fw | CGTGCGAUAAGGAGTTTGGCGCCCGTT |
| PR-15522 | PrExp_rv | ATGACAGAUTGCTGTAGATATGTCTTGT |
| PR-15528 | PrGPD1_rv | ATGACAGAUTGTTGATGTGTGTTTAATTCAAGAATG |
| PR-15529 | PrGPD1_forfusion_fw | AGCTACTGAUGACGCAGTAGGATGTCCTGCACGG |
| PR-15605 | <-PrExp_forfusion_U1_fw | ATCAGTAGCUAAGGAGTTTGGCGCCCGTT |
| PR-15606 | <-PrExp_for fusion_U1_rv | ACCTGCACUTGCTGTAGATATGTCTTGT |
| PR-15788 | PrtRNAGly_fw | taaccaaccUgcgccgacccggaatcgaac |
| PR-16706 | ku70_up _fw | CGTGCGAUATGGTGTGCCAGGAGGTGG |
| PR-16708 | ku70_down _fw | AGTGGCCUTTTCAAAAAGCGGCGGTTCG |
| PR-16709 | ku70_down _rev | CACGCGAUTTGGGCTTGGGGCACTTCTG |
| PR-16713 | PrGPD_rev | ATGTTGAUGTGTGTTTAATTCAAGAATG |
| PR-16714 | DsdAsyn _fw | ATCAACAUGGAAAACGCTAAAATGAAC |
| PR-16715 | DsdAsyn _rev | AACAGAAGUTAACGGCCTTTTGCCAGATATTG |
| PR-16716 | TLip_fw | ACTTCTGTUCGGAATCAACCTCAAGGTTAAC |
| PR-17826 | ku70_up (int vector)_rev | aagcgttgcacgUCTAGGGAGGCACATCTAAAC |
| PR-18214 | PrTEFintron_USER_rv | AGTACTGCAAAAAGUGCTG |
| PR-18931 | PrTEF1->_forfusion_U2_fw | AGCTACTGAUAGAGACCGGGTTGG |
| PR-21805 | gRNA1_SDH5_sense | AGCTGGCCGAGAACAAGGAGGTTTTAGAGCT |
| PR-21806 | gRNA1_SDH5_antisense | CTCCTTGTTCTCGGCCAGCTTAACCAACCT |
| PR-21807 | gRNA2_SDH5_sense | CCAGGCCAACATCAACCCTGGTTTTAGAGCT |
| PR-21808 | gRNA2_SDH5_antisense | CAGGGTTGATGTTGGCCTGGTAACCAACCT |
| PR-21809 | KO_ACH1_UP_fw | GTCCTTCTCCATGTGTATT |
| PR-21810 | KO_ACH1_UP_rv | AGGCCACUTGTGTGAGATGGGGTAGT |
| PR-21811 | KO_ACH1_DW_fw | AGTGGCCUCCTATTGATGTTCCTGATC |
| PR-21812 | KO_ACH1_DW_rv | GGCGGGGAAATTACACTA |
| PR-21815 | gRNA2_ACH1_sense | CATGTGGACAATCTCAGGGGGTTTTAGAGCT |
| PR-21816 | gRNA2_ACH1_antisense | CCCCTGAGATTGTCCACATGTAACCAACCT |
| PR-21845 | gRNA3_SDH5_sense | CTTGATGTTGTCGTTTCGGGGTTTTAGAGCT |
| PR-21846 | gRNA3_SDH5_antisense | CCCGAAACGACAACATCAAGTAACCAACCT |
| PR-21847 | gRNA4_SDH5_sense | GCTGGCCGAGAACAAGGAGCGTTTTAGAGCT |
| PR-21848 | gRNA4_SDH5_antisense | GCTCCTTGTTCTCGGCCAGCTAACCAACCT |
| PR-21915 | TPOT1->_forfusion_U3_fw | ACTTACAAUATGTACATACAAGATTATTTATAG |
| PR-21916 | TPOT1->_forfusion_U3_rv | ATCAGTAGCUACCGCTCGACAACCTCCC |
| PR-22053 | ICL_forfusionPTefin_U2_fw | ACTTTTTGCAGTACUAACCGCAGCTTCGAACACGATTCATC |
| PR-22054 | ICL_forfusionPTefin_U2_rv | CACGCGAUTTAAAACTGGTTCTCGGT |
| PR-22055 | MLS_U1_fw | AGTGCAGGUGCCACA ATGGGGCCTGGTCTGCCG |
| PR-22056 | MLS_U1_rv | CGTGCGAUTTAGAGCTTGGAAGAGCT |
| PR-22057 | MDH_U2_fw | ATCTGTCAUGCCACAATGTTCCAACCCCGAGTT |
| PR-22058 | MDH_U3_rv | ATTGTAAGUTTAAGGGTTCTGCTTGAC |
| PR-22059 | scPYC1_U1_fw | AGTGCAGGUGCCACAATGTCGCAAAGAAAATTCG |
| PR-22060 | scPYC2_U2_fw | ATCTGTCAUGCCACAATGAGCAGTAGCAAGAAATTG |
| PR-22061 | AsPCK_U2_fw | ATCTGTCAUGCCACAATGACCGACTTGAAC |
| PR-22337 | SCS_U1_fw | AGTGCAGGUGCCACAATGTTTTCGCGAATTGC |
| PR-22338 | SCS_U1_rv | CGTGCGAUTTAAAGAGGCAGCTCAAA |
| PR-22339 | KGDH_U2_fw | ATCTGTCAUGCCACAATGCTCAGACACGCTTT |
| PR-22340 | KGDH_U2_rv | ATTGTAAGUTTAGGCCTTATCCTCCTG |
| PR-22341 | YlPYC_U2_fw | ATCTGTCAUGCCACAATGTCTCCCCCCGTCAA |
| PR-22342 | YlPYC_U2_rv | ATTGTAAGUCTAAGCCTTAGGGCCGGC |
| PR-22343 | YlPCK_U2_fw | ATCTGTCAUGCCACAATGTCCAACGTTCCTGA |
| PR-22344 | YlPCK_U2_rv | ATTGTAAGUTTAAGCCCGCACAATCTT |
| PR-22345 | scPCK_U2_fw | ATCTGTCAUGCCACAATGTCCCCTTCTAAAATGA |
| PR-22346 | scPCK_U2_rv | ATTGTAAGUTTACTCGAATTGAGGACC |
| PR-22347 | AsPYC_U2_fw | ATCTGTCAUGCCACAATGACCGACCTGAACAAG |
| PR-22348 | AsPYC_U2_rv | CACGCGAUTTAGGCCTTGGGACCAGCGC |
| PR-22365 | SpMae1_U2_fw | ATCTGTCAUGCCACAATGGGTGAACTCAAGGAAATC |
| PR-22390 | KO_SDH5_UP_U_fw | CGTGCGAUGCTGAGATGGACTAGTGT |
| PR-22391 | KO_SDH5_UP_U_rv | AGCTGTTCUTTTAGTCGGTTCAAAGCTC |
| PR-22392 | KO_SDH5_DW_U_fw | AGTGGCCUGTGTGATTGTGATCGAAAC |
| PR-22393 | KO_SDH5_DW_U_rv | CACGCGAUGAGAGTCTGGAATCTTCTATG |
| PR-22690 | KO_PSDH1_repair_UP_fw | CGTGCGAUATGGTGCGAATGGGTGT |
| PR-22691 | KO_PSDH1_repair_UP_rv | AGCTGTTCUCCACGCGTACAGCCAAA |
| PR-22693 | KO_PSDH1_repair_gRNA1_Dw_rv | CACGCGAUGACAGCTGGAGGAAGAT |
| PR-22694 | KO_PSDH1_repair_gRNA2_Dw_fw | AGTGGCCUCATCCAGACAACTATTCTC |
| PR-22698 | gRNA2_PSDH1_sense | CTGGATGATTTACTGCAGAGGTTTTAGAGCT |
| PR-22699 | gRNA2_PSDH1_antisense | CTCTGCAGTAAATCATCCAGTAACCAACCT |
| PR-671 | ScPYC1_U1_rv | CGTGCGAUTCATGCCTTAGTTTCAACAG |
| PR-673 | ScPYC2_U2_rv | CACGCGAUTTACTTTTTTTGGGATGGG |
| PR-7016 | AsPCK_U2_rv | CACGCGAUTTAAGCTTTAGGACCAGCAC |
| PR-7030 | SpMae1_U2_rv | CACGCGAUTTAAACGCTTTCATGTTCAC |
| **Sequencing Primers** | | |
| PR-10594 | for gRNA expression cassette | CATGGCGGCCGCGAATGC |
| PR-14441 | PrGPD_seqcheck_fw | GTCTGCATCGCCGGCTC |
| Pr-14617 | Gene1_check_rev | TATCCCTGTGTTGAATC |
| PR-14618 | PrTefintron_check_fw | GGCTTTGAGCACACG |
| PR-14619 | Gene2_check_rv | TATCGACCCAGTTAGC |
| PR-15588 | PrEXP_seqcheck_fw | GCCCGTCTTCCCTTGCTAAC |
| PR-22374 | ScPYC2_check_fw | CCAAATGGTATCGTCACAA |
| PR-22375 | ScPYC2_check_rv | CGGTTTCATAGTACCTGC |
| PR-22382 | YlPYC cloning check | GAATCCGAGGAGTCAAGA |
| PR-22388 | AsPYC_check_fw | AACTTCAAGGCCGACTTC |
| PR-22389 | AsPYC_check_rv | GCCTTCATTCGCTCGAC |
| **Yeast genotype primers** | | |
| PR-14832 | IntD_1_check_fw | ACTGGTGGCTACAAATGAAG |
| PR-14834 | IntC_2_check_fw | AAAGTCCAACAGATTTCAGC |
| PR-14835 | IntE_3_check_fw | CACGCACGCCATTCTATAAG |
| PR-14838 | IntC_3_check_fw | AAACCTGTCGTATGAGCTAC |
| PR-8859 | TPex20 _fw | aagtgtggatggggaagtgag |

**Supplementary Sequences**

Sequences of synthetic genes

>AsPYC

ATGACCGACCTGAACAAGCTGGTGAAGGAACTGAACGACCTGGGCCTGACCGACGTGAAGGAAATCGTGTACAACCCCTCTTACGAGCAGCTGTTCGAGGAAGAGACTAAGCCCGGCCTCGAGGGCTTCGACAAGGGCACCCTGACCACTCTGGGCGCCGTGGCCGTGGACACCGGCATCTTCACCGGACGATCTCCCAAGGACAAGTACATCGTGTGCGACGAGACTACCAAGGACACCGTGTGGTGGAACTCTGAGGCCGCCAAGAACGACAACAAGCCCATGACTCAAGAGACTTGGAAGTCCCTGCGAGAGCTGGTGGCCAAGCAGCTGTCTGGCAAGCGACTGTTCGTGGTCGAGGGCTACTGCGGCGCCTCTGAGAAGCACCGAATCGGCGTGCGAATGGTGACCGAGGTGGCCTGGCAGGCCCACTTCGTGAAGAACATGTTTATTCGACCCACCGACGAGGAACTCAAGAACTTCAAGGCCGACTTCACCGTGCTGAACGGCGCCAAGTGCACTAACCCCAACTGGAAGGAACAGGGCCTGAACTCCGAGAACTTCGTGGCCTTCAACATCACCGAGGGCATCCAGCTGATCGGCGGCACCTGGTACGGCGGCGAGATGAAGAAGGGCATGTTCTCTATGATGAACTACTTCCTGCCTCTGAAGGGCGTCGCCTCTATGCACTGCTCTGCCAACGTCGGCAAGGACGGCGACGTGGCCATCTTCTTCGGCCTGTCTGGAACCGGCAAGACTACCCTGTCTACTGACCCCAAGCGACAGCTGATTGGTGACGACGAGCACGGCTGGGACGAGTCTGGCGTGTTCAACTTCGAAGGCGGCTGCTACGCCAAGACCATCAACCTGTCTCAAGAGAACGAGCCCGACATCTACGGCGCCATCCGACGAGATGCCCTGCTCGAGAACGTGGTGGTGCGAGCCGACGGCTCTGTGGACTTCGACGACGGATCTAAGACCGAGAACACCCGAGTGTCTTACCCCATCTACCACATCGACAACATCGTGCGACCCGTGTCTAAGGCCGGCCACGCTACCAAGGTGATCTTCCTGACCGCCGACGCCTTCGGCGTGCTGCCTCCTGTGTCTAAGCTGACCCCTGAGCAGACCGAGTACTACTTTCTGTCTGGCTTCACCGCCAAGCTGGCCGGCACCGAGCGAGGCGTGACCGAGCCTACTCCTACCTTCTCTGCCTGCTTCGGCGCTGCTTTCCTGTCTCTGCACCCTATCCAGTACGCCGACGTGCTGGTCGAGCGAATGAAGGCCTCTGGCGCTGAGGCCTACCTGGTGAACACCGGCTGGAACGGCACCGGCAAGCGAATCTCTATTAAGGACACCCGAGGCATCATCGACGCCATCCTGGACGGCTCTATCGAGAAGGCCGAGATGGGAGAGCTGCCCATCTTCAACCTGGCTATCCCCAAGGCTCTGCCCGGCGTGGACCCCGCCATTCTGGACCCTCGAGACACCTACGCCGACAAGGCCCAGTGGCAGGTCAAGGCTGAGGACCTGGCCAACCGATTTGTGAAGAACTTTGTGAAGTACACTGCTAACCCCGAGGCCGCTAAGCTGGTCGGCGCTGGTCCCAAGGCCTAA

>AsPCK

ATGACCGACTTGAACAAGTTGGTCAAAGAATTGAACGATTTGGGTTTGACCGACGTCAAAGAAATCGTTTACAATCCATCCTACGAACAATTATTCGAAGAAGAAACTAAGCCAGGTTTGGAAGGTTTTGATAAGGGTACTTTGACTACTTTGGGTGCTGTTGCTGTTGATACTGGTATTTTTACTGGTAGATCCCCAAAGGATAAGTACATCGTTTGTGACGAAACTACCAAGGATACTGTTTGGTGGAATTCTGAAGCTGCTAAGAATGATAACAAGCCAATGACTCAAGAAACCTGGAAGTCTTTGAGAGAATTGGTTGCTAAACAATTGTCCGGTAAGAGATTATTCGTTGTCGAAGGTTATTGTGGTGCTTCTGAAAAACATAGAATCGGTGTTAGAATGGTTACCGAAGTTGCTTGGCAAGCTCATTTTGTCAAGAATATGTTCATCAGACCAACCGACGAAGAATTGAAGAATTTCAAGGCTGATTTCACCGTTTTGAACGGTGCTAAATGTACTAATCCAAACTGGAAAGAACAAGGTTTGAACAGTGAAAACTTCGTCGCTTTCAACATTACCGAAGGTATTCAATTGATTGGTGGTACTTGGTATGGTGGTGAAATGAAGAAAGGTATGTTCTCCATGATGAACTACTTTTTGCCATTGAAGGGTGTTGCTTCTATGCATTGCTCTGCTAATGTTGGTAAAGATGGTGATGTTGCCATTTTCTTTGGTTTGTCTGGTACTGGTAAAACCACTTTGTCTACTGATCCTAAGAGACAATTGATCGGTGATGATGAACACGGTTGGGATGAATCTGGTGTTTTTAACTTTGAAGGTGGTTGTTACGCCAAGACCATTAACTTGTCTCAAGAAAACGAACCAGATATCTACGGTGCCATTAGAAGAGATGCTTTGTTGGAAAACGTTGTTGTTAGAGCTGATGGTTCCGTTGATTTTGATGATGGTTCTAAGACCGAAAACACTAGAGTTTCTTACCCAATCTACCACATCGATAACATCGTTAGACCAGTTTCTAAAGCTGGTCATGCTACCAAGGTTATTTTCTTGACTGCTGATGCTTTTGGTGTTTTGCCACCAGTTTCAAAATTGACTCCAGAACAAACCGAATATTACTTCTTGTCTGGTTTCACTGCTAAGTTGGCTGGTACTGAAAGAGGTGTTACTGAACCTACTCCAACTTTTTCTGCTTGTTTTGGTGCTGCTTTCTTGTCCTTGCATCCAATTCAATACGCAGATGTTTTGGTTGAAAGAATGAAGGCTTCTGGTGCTGAAGCTTACTTGGTTAATACTGGTTGGAATGGTACAGGTAAGAGAATCTCTATTAAGGACACCAGAGGTATTATCGATGCCATTTTGGATGGTTCCATCGAAAAAGCTGAAATGGGTGAATTGCCAATTTTCAACTTGGCTATTCCAAAAGCTTTGCCAGGTGTTGATCCAGCTATTTTAGATCCAAGAGATACCTATGCTGATAAGGCACAATGGCAAGTTAAGGCTGAAGATTTGGCTAACAGATTCGTTAAGAACTTCGTTAAGTACACCGCTAATCCAGAAGCTGCAAAATTGGTTGGTGCTGGTCCTAAAGCTTAA

**References**

Angerer, H., Radermacher, M., Makowska, M., Steger, M., Zwicker, K., Heide, H., et al. (2014). The LYR protein subunit NB4M/NDUFA6 of mitochondrial complex I anchors an acyl carrier protein and is essential for catalytic activity. Proc. Natl. Acad. Sci. 111, 5207–5212. [doi:10.1073/pnas.1322438111](https://doi.org/10.1073/pnas.1322438111)

Borodina, I., Kildegaard, K.R., Jensen, N.B., Blicher, T.H., Maury, J., Sherstyk, S., [Schneider, K](https://www.ncbi.nlm.nih.gov/pubmed/?term=Schneider%20K%5BAuthor%5D&cauthor=true&cauthor_uid=25447643)., et al. (2015). Establishing a synthetic pathway for high-level production of 3-hydroxypropionic acid in *Saccharomyces cerevisiae* via β-alanine. Metabol. Eng. 27, 57–64. doi:10.1016/j.ymben.2014.10.003.

Holkenbrink, C., Dam, M.I., Kildegaard, K.R., Beder, J., Dahlin, J., Doménech Belda, D., et al. (2017). EasyCloneYALI: CRISPR/Cas9-based synthetic toolbox for engineering of the yeast *Yarrowia lipolytica*. Biotechnol. J. 13, 9. doi:10.1002/biot.201700543.

Kildegaard, K.R., Adiego-Pérez, B., Doménech Belda, D., Khangura, J.K., Holkenbrink, C., Borodina, I. (2017). Engineering of *Yarrowia lipolytica* for production of astaxanthin. Synth. Syst. Biotechnol. 2, 287–294. [doi:10.1016/j.synbio.2017.10.002](https://doi.org/10.1016/j.synbio.2017.10.002)

Marella, E.R., Dahlin, J., Dam, M.I., Horst, J.T, Christensen, H.B., Sudarsan, S., et al. (2019). A single host fermentation process for the production of flavor lactones from non-hydroxylated fatty acids. Metabol. Eng. In Press.

Stovicek, V., Borja, G.M., Forster, J., Borodina, I. (2015). EasyClone 2.0: expanded toolkit of integrative vectors for stable gene expression in industrial *Saccharomyces cerevisiae* strains. J. Ind. Microbiol. Biotechnol. 42, 1519–1531. doi:[10.1007/s10295-015-1684-8](https://dx.doi.org/10.1007%2Fs10295-015-1684-8)
